# Development and Testing of a Multimedia Internet-Based System for Fidelity and Monitoring of Multidimensional Treatment Foster Care

**DOI:** 10.2196/jmir.2034

**Published:** 2012-10-16

**Authors:** Edward G Feil, Peter G Sprengelmeyer, Betsy Davis, Patricia Chamberlain

**Affiliations:** ^1^Oregon Research InstituteEugene, ORUnited States; ^2^OSLC Community ProgramsEugene, ORUnited States; ^3^Oregon Social Learning CenterEugene, ORUnited States

**Keywords:** Mental health, fidelity, clinical supervision, multidimensional treatment foster care, multimedia

## Abstract

**Background:**

The fields of mental health, child welfare, and juvenile justice are jointly faced with the challenge of reducing the prevalence of antisocial behavior among adolescents. In the last 20 years, conduct disorders have moved from being considered intractable difficulties to having complex but available solutions. The treatments for even long-standing offending behavior among adolescents are now well documented and supported by a growing and compelling body of evidence. These empirically validated interventions are being widely disseminated, but the replication of the results from clinical trials in community settings has yet to be documented. The treatments, which produced impressive effects in a research context, are difficult to replicate without intensive monitoring of fidelity by the developers. Such monitoring is a barrier toward adoption; as the distance between the adopter and developer increases, so does cost. At the same time, states, communities, and agencies are under increasing pressure to implement those intervention services that have been shown to be most effective. The use of the Internet offers a potential solution in that existing reporting and data collection by clinicians can be subject to remote supervision. Such a system would have the potential to provide dissemination teams with more direct access to higher-quality data and would make adopters more likely to be able to implement services at the highest possible conformity to research protocols.

**Objective:**

To create and test such an innovative system for use with the Multidimensional Treatment Foster Care (MTFC) program, which is an in-home treatment (alternative to a residential- or group-home setting) for antisocial youths. This research could advance the knowledge base about developing innovative infrastructures in community settings to disseminate empirically validated treatments.

**Methods:**

The fidelity system was used and reviewed by parent and professional users: 20 foster parent users of the Parent Daily Report function, 9 professional MTFC program supervisors, and 4 MTFC consultants. All participants rated the system’s ease of use, quality of the website, and observational videos recorded at agency meetings. In addition, foster parents entered data on child behavior.

**Results:**

All professionals and foster parents rated the system as very easy to use. We found particularly high levels of use by parents. Professionals rated the computer-collected videos of clinical meetings as being of high quality and easily codeable.

**Conclusions:**

The project developed a user-friendly and secure Web-based system using state-of-the-art computer-based protocols for recording questionnaire and observational data generated by community-based MTFC staff and foster parents, with positive satisfaction and utilization results.

## Introduction

In the recent past, adolescent delinquent behavior and conduct disorder generally were considered to be intractable problems for which the only interventions were removal from the community and individual-based sanctions (eg, detention and community service work). Since then, several treatment and prevention approaches have been developed (eg, Multisystemic Therapy, Functional Family Therapy, and Multidimensional Treatment Foster Care [MTFC] [1]), each of which have proven to be extremely effective in reducing the number and seriousness of offenses for individual youths, in linking the problematic youths into their families and communities, and in preventing placements for youths in more restrictive settings (eg, boot camps and secure locked facilities). Such restrictive placements are both more costly and correlated with a much higher rate of entry into adult corrections than are community placements [2].

### Empirically Validated Treatments

Along with the efforts to examine capacity expansion and dissemination, there has been a consistent increase in the call for communities to adopt empirically validated practices from federal programs, state policies, and local advocacy. Some of the necessary infrastructure for dissemination has been or is being developed. The programs identified as validated have been manualized, a cadre of trainers has been established, and the listed programs shown to be effective have been widely publicized. However, an ongoing problem has been that without extensive direct support from the program developers, the community implementations of these practices remain substantially less effective than they were during the efficacy trials [3]. There is likely a variety of reasons for this decrement in the effectiveness as these interventions are disseminated into communities: for instance, lack of fidelity to core intervention practices, attempting to expand the services beyond the population shown to be positively affected, and limits of the original efficacy studies (eg, restricted clinical population, guaranteed funding for services, and highly trained interventionists) have all been suggested as increasing the treatment effects in efficacy trails and limiting the effectiveness of the interventions when applied in the community (real world).

### Multidimensional Treatment Foster Care

MTFC [1] is the only empirically validated intervention that has been demonstrated to be effective with youth who have been removed from their homes because of their extensive antisocial behavioral histories. MTFC has been supported with the results of an independent cost-benefit analysis by the Washington State Public Policy group [4] and evidence from several randomized trials (eg, [5-7]). In addition, the MTFC model has been selected as 1 of 10 evidence-based national model programs (The Blueprints Programs [8]) in the United States by the Office of Juvenile Justice and Delinquency Prevention and as 1 of 9 national Exemplary and Promising Safe, Disciplined, and Drug-Free Schools model programs. MTFC was also highlighted in two US Surgeon General reports [9,10] and was selected by the Center for Substance Abuse Prevention and Office of Juvenile Justice and Delinquency Prevention as an Exemplary I program for Strengthening America’s Families [11]. Early study participants have now been followed up as much as 10 years after initial MTFC participation, and treatment effects appear to be sustained for some time following the intervention [12].

The basic MTFC model involves placing an adolescent in a well-trained and supervised foster home where MTFC parents have undergone 20 hours of preservice orientation and training after having been certified by the state to be foster parents. Experienced case managers with small caseloads (8-10 MTFC families each) maintain daily contact with MTFC parents to provide ongoing consultation, support, and crisis intervention. Case managers coordinate all aspects of the youth’s placement, including ensuring that interventions in the family and individual therapy and with the skills trainer are targeting key behaviors. Basic components of MTFC are (1) daily telephone contact (during the workweek) with MTFC parents using the Parent Daily Report (PDR) measure [13]), (2) participation of MTFC parents in a weekly group supervision and support meeting led by the case manager, (3) implementation by MTFC parents of an individualized daily point-and-level program for the adolescent placed with them, (4) individual therapy for the participating adolescent, (5) family therapy (for aftercare resource) focusing on parent management strategies, (6) close monitoring of school attendance, performance, and homework completion, (7) case management to coordinate the interventions in the MTFC, family, peer, and school settings, (8) round-the-clock on-call program staff availability to MTFC and biological parents, (9) psychiatric consultation as needed, and (10) weekly clinical team meetings where the multiple intervention components are coordinated.

### Barriers to Adoption and Dissemination

Like many of the empirically validated treatments, MTFC requires a shift in the manner in which traditional mental health, juvenile justice, and child welfare services are conducted. Staffing, funding, and reporting are often quite different within MTFC from how these functions operate in the broader community service organizations that may choose to replicate the MTFC model. For example, several elements of the MTFC intervention program (eg, PDR data collection or weekly staff meetings) do not fall under typical Medicaid reimbursement codes. Most service agencies also do not have established infrastructures to recruit, train, and certify foster care providers. Therefore, the practical implementation of the MTFC model can meet with several practical barriers early in the implementation process.

Because of these barriers, early efforts to disseminate MTFC were difficult and not satisfactory. An organization, separate from the direct research and treatment endeavors, was created (in 2001) with the sole purpose of disseminating the MTFC program: TFC Consultants, Inc. TFC Consultants helped develop structured program manuals, wrote training materials, scripted training procedures, and recruited clinical and foster parent trainers. There are currently 30 organizations in the United States, Canada, the United Kingdom, Sweden, and Norway working at various stages of program development. It has been clear during this process that fidelity standards and processes were also needed to track the process of program implementation in addition to the functional elements of the program. For example, an Internet-based PDR data-gathering tool was developed to allow MTFC site consultants immediate access to behavioral reports of program youths. Reporting instruments were also developed for the weekly telephone consultations with the implementation sites, for quarterly reporting of fidelity, and most recently for site certification (by a separate research organization, the Center for Research to Practice). While MTFC has tracked fidelity in this way, other empirically validated programs either offer no tracking of program fidelity after the initial training or offer continual quality improvement support without any method to measure or reduce the costs of this support. The position of the MTFC developers has been that there must be a balance between not requiring service organizations to bear ongoing consultation expenses and needing to maintain fidelity for those running MTFC programs. For MTFC, the developers hold that the optimal balance involves giving implementation sites clear feedback about their position relative to fidelity standards during training, and then supporting a certification process where organizations can demonstrate that the program is meeting fidelity standards and is no longer in need of external support.

We present our findings on the development and the feasibility evaluation of an Internet-based treatment fidelity monitoring system (ITFMS) funded by a Phase I Small Business Technology Transfer grant from the US National Institute of Mental Health (#R42MH075174). The ITFMS has three primary components: (1) an agency-tailored secure MTFC website with multiple levels of access restrictions for data storage, and display of reports (both graphical and tabular) and observations for dynamic feedback, (2) computer-mediated forms for clinical (eg, daily reporting of child behavior) and supervisory activities (eg, program tasks such as therapy appointments and clinical meetings) tailored to the respondent with Internet-based and automated phone-based response formats, and (3) computer-mediated video observation of clinical meetings.

## Methods

### Development of User-Interface Programming

Using our experience with previous Internet-based interventions for parenting [14-17], type 2 diabetes [18,19], and smoking cessation [20], we developed a graphic user interface that is appealing and easy to navigate. The programming included the use of the PHP scripting language for the generation of dynamic content that is read from a MySQL database. The combination of Hypertext Markup Language (HTML) and cascading style sheets was used to structure and lay out the elements of a page. The development phase of the Phase I project involved designing and programming the video capture software, delineating a protocol for securely transferring the video-recorded observations, developing the database, and programming a secure website for data entry and display. The software (eg, automated video recording) was menu driven using the mouse and icons to minimize the need for keyboarding skills. The video recording software was developed using Flex (Adobe Systems Inc, San Jose, CA, USA) to create a Flash-based object to securely stream it to the server for review.

We captured the videos of clinician–foster parent interactions in clinical meeting settings to a digital format using a preloaded video capture Flash-based object. This produced high-quality, low-data-rate movies that take up minimal bandwidth. We captured the video at a resolution of 464 × 346 ppi, 15 fps, about 40–45 KB/s. As the MTFC intervention makes extensive use of video observation (each week, a clinical team supervision and a foster parent support group meeting, each lasting 2 hours, are recorded and transferred to the site consultant for review), the central task of developing the fidelity management system for the computer was to use videos in less performance-intensive elements (ie, reducing video resolution to improve performance in Flash using Adobe Flex).

### Encryption and Internet Security Development

We used state-of-the-art security protocols in all data collection and monitoring activities, as is used for e-commerce, using secure sockets layer data encryption technology. The website was established within a suite of servers housed in an existing secure network and with independent firewall security. The website is accessible via the Internet with username and password protection. Once admitted to the site, each user has a hierarchically assigned set of permissions that allow the user to view or edit, or both, only that information that is necessary for his or her job functions. So, an individual foster parent may be allowed to enter PDR data, but would be able to do so only for the youth in his or her home (this can be reassigned to permit respite) and would not be able to alter or delete any data. Alternatively, a program supervisor has access to the data for the youth in his or her program. A consultant has access to data across programs assigned to him or her. PDR data are reviewed at weekly meetings between the foster parents and program supervisor to monitor accuracy.

### Structure of the ITFMS

**Figure 1:**
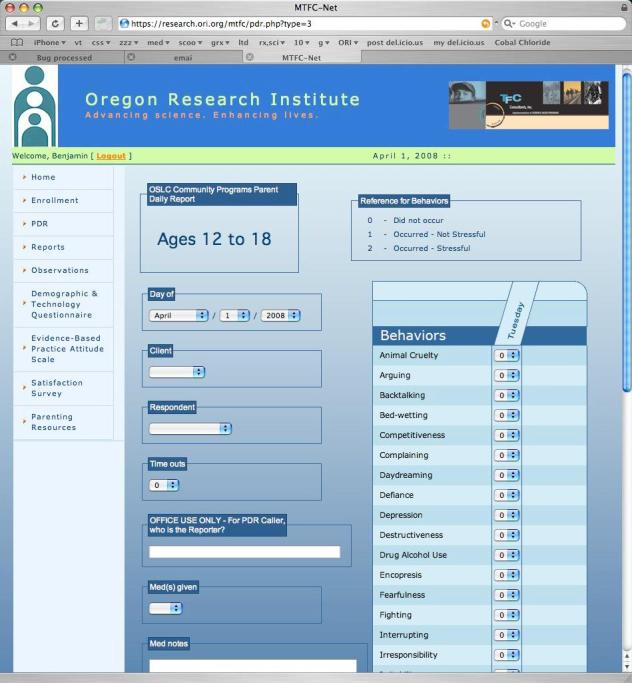
Parent Daily Report form in the Internet-based treatment fidelity monitoring system.

**Figure 2:**
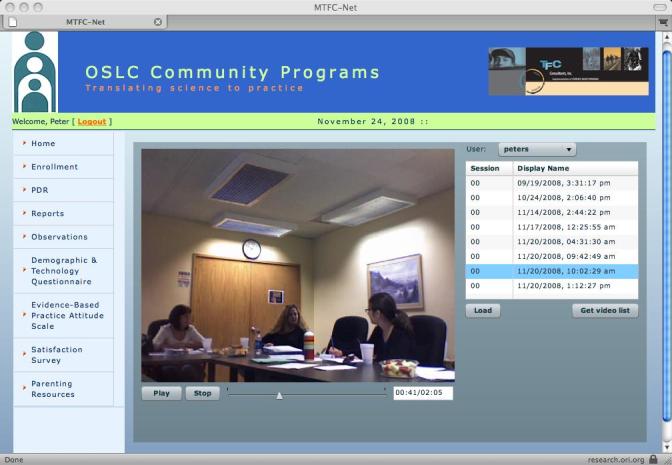
Video reviewing screen showing a sample video of a clinical meeting.

The ITFMS system contains 9 left-hand, navigational menu options, 6 of which were developed. First, the *Home *option provides a welcome to the system and a brief description of what the system will provide the user. Second, the *Enrollment *option provides 3 submenus for (1) Enroll Agency, which serves as the initial system setup, including information such as agency name, Web and street address, MTFC certification contact person information, the date of MTFC program initiation at the agency, and a place for specific MTFC role positions to be identified, (2) Enroll Client, which contains a place for first name only, age group related to MTFC programs (3–6 years, 7–11 years, and 12–18 years), and the month and year the client began the program, and (3) User, which includes a place for name, address, phone number, email address, and a username and password to get into the ITFMS system for PDRs (these pieces of information were designed to allow for the entry of pieces of data necessary for successful completion of the MTFC certification process). Third, the *PDR *option provides 3 submenus reflecting MTFC age groups (3–6 years, 7–11 years, and 12–18 years), to which a foster parent has access based on the developmental level of the youth in his or her home. The list of available options is created at each log-in. Beside each item listed (eg, animal cruelty) is a pull-down menu with options 0, 1, and 2 (0 = behavior did not occur; 1 = behavior did occur and was not stressful; and 2 = behavior did occur and was stressful) from which to select an answer (see Figure 1). Fourth, the *Reports *option provides a client pull-down menu that allows agencies and consultants to select the foster adolescent for whom they wish to view PDR and Behavioral Points summaries, as well as a date window from which a specific week can be selected through the use of a calendar image. Through this report system, a week’s worth of PDR and Behavioral Points data, entered through any system (Internet, phone, or agency caller), can be displayed by day of the week, indicating for each day the number of Time Outs Given, Behavioral Points Earned for good behavior, Behavioral Points Lost for inappropriate behavior, Total PDR Behaviors Reported as occurring and stressful, Total Intensity of these behaviors, the Interviewer and Respondent for each day, as well as whether any medications were given. Fifth, the *Observation *option provides 2 submenu options for recording or playing. When each option is selected, a video screen appears, with the option to record video or play video. Additionally, on the play video page, a bland tabular window is presented with a pull-down menu that allows users to select the type of videos they wish to view, whether clinical or foster parent meetings. When type of video is selected, the rows in the tabular fields are filled in with a list of sessions recorded and, for each session, a display name for that video file. When selected, the video file is retrieved from the database and loaded into the video screen for reviewing. This retrieval system allows multiple users (eg, clinicians and consultants) to simultaneously stream recorded video from multiple locations for direct supervision (see Figure 2).

Within the PDR system, foster parents are allowed to decide whether they wish to report their PDR data using the Internet-based system, using the phone-based interface, or having an agency caller contact them. Having options provides parents with convenience in terms of their time and comfort with technology. MTFC practice is to have an individual caller contact each foster parent daily and ask a series of standardized questions about the youth’s behavior during the preceding 24 hours. With the Web-based system, the respondent simply enters the PDR information directly, using a series of drop-down menus (as described above). With the phone-based system, the respondent calls into the system, enters a pass code (that authorizes access to the system and links the caller to a specific youth), and then is prompted with a standard series of behaviors to report using the phone’s keypad relative to whether the behavior occurred. All 3 formats collect the same data, and data are stored in the same format within the ITFMS, but the use of the human caller creates scheduling limitations and an additional level of potential data-entry errors. In addition, the systems are set to allow multiple systems to be used, such that a foster parent who does not enter data by a given time might be provided with an automated call. The figures show pages from the ITFMS system, one reflecting the video recording of an agency clinical staff meeting (Figure 2), as viewed by consultants, and the other reflecting the PDR page wherein foster parents provide their daily behavior ratings and the number of behavior points given or taken away (Figure 1).

### Measures

We assessed the ITFMS with measures administered to parent and professional users. Computer-administered measures included demographics, child behavior, and satisfaction. All agency supervisors and foster parents rated the ease of use of the system on a 7-point Likert-type scale from 1 = very difficult to 7 = very easy. MTFC consultants rated video clips relative to audio and video quality on 5-point scales anchored by 1 = impossible, 3 = codeable, and 5 = easy to code. User tracking measures of log-ins and time on website were collected automatically by the server. Data were collected from 20 foster parent PDR users, 9 professional MTFC program supervisors, and 4 MTFC consultants, who rated the quality of observational videos recorded from agency meetings. The foster parents completed PDR questionnaires on the behavior of 20 foster children in their care. As noted above, encryption and permissions allowed foster parents to view only their own data. Staff were allowed to view PDR data only on children on their caseload.

## Results

### Participants

Foster parents were on average 33.2 years old, had 3.6 people in their household, and had a median family income range of US $40,000–$45,000; 14 (70%) had completed high school, with 6 (30%) reporting attending college; and 15 (75%) of the parents reported working outside of the home, with 4 (20%) indicating that were not working, by their own choice. All of the participating parents were white, with 1 parent (5%) reporting their ethnicity as Hispanic/Latino; 18 (90%) of the parents reported being married or living together with another as a couple. Of the participating parents, all reported having a working telephone, and 18 (90%) indicated they had a computer at home. Parents reported high levels of computer use, with 16 (80%) indicating more than 10 hours per week, 12 (60%) indicating more than 20 hours per week, and only 4 (20%) reporting 5 or less hours per week. Of the parent PDR users, only 4 (20%) had computer access through their work, and so computer use was predominantly at home.

Professional participants, both agency and consultant, were on average 36.3 years old and had a median family income range of US $50,000–$60,000; 11 (85%) had completed a postgraduate degree. A total of 10 (77%) of the professionals reported they were white, with the remainder declining to state their race; 1 (11%) of the professionals reported being Hispanic/Latino. In addition, 10 (91%) of the professionals reported being married or living together with another as a couple. All professionals reported owning a phone and having a computer at home. Much like the parents, professionals reported high levels of computer use, with 9 (69%) reporting more than 20 hours per week and only 1 (8%) reporting 5 or less hours per week; 11 (85%) of the professionals reported having access to a computer at work.

### Ease of Use

All agency supervisors and foster parents rated the ease of use of the system as very easy to use. When asked if they would recommend the system to other staff or parents, on a 7-point Likert-type scale from strongly recommend to strongly not recommend, all responses were strongly recommend (score of 7) or recommend (6). All individuals further reported a timesaving benefit on the PDR behavioral reports, either for entering data (parents) or for viewing summaries (consultants), relative to the typical PDR method (ie, PDR caller). Comments were overwhelmingly positive. Example comments included “I definitely enjoy this better than a caller who calls me at home! Thanks!” (parent); “The system seems very well designed and is easy to look at and find what you are looking for!” (parent); “It would be interesting to compare the data that we get from a live person asking questions” (staff); “The whole process is very simple to use” (staff); “Very good support, thanks!!;” and “I like doing PDR on my own time schedule.”

### ITFMS Utilization

For both the PDR (parents) and Report (supervisor) functions, the Phase I trial was designed to last 1 week. Thus, submission of 7 PDRs via the Web-based or automated telephone PDR system indicated completion of the trial. All 20 parents completed the trial, some providing data for multiple children in the home. Reports were submitted for 28 foster children, and even though the trial was intended for week, a high number of parents continued on past the 7-day trial. Some parents enjoyed using the Web-based entry because it allowed them to give their daily reports at times convenient to them, rather than having to schedule a call with staff within their busy schedules. As many states require foster parents to demonstrate sources of income outside of child welfare system support payments, this flexibility is increasingly important. A total of 12 parents completed more than 14 PDRs, with 6 families completing at least 21. Utilization was particularly high among 2 parents, who completed 145 and 176 PDRs, respectively, on their children. A total of 667 PDRs were completed during the trial. In terms of supervisor program utilization, all supervisors reported using the Web-based PDR system to view weekly behavior summaries. In addition, 4 (36%) reported using Web-based viewing of meeting observations and 7 (64%) reported using the Web-based enrollment and administrative features.

### Satisfaction

Of the 9 supervisors, 8 (89%) found the overall system very easy to use, with all 9 indicating the system was either fairly easy or very easy to use. Supervisors also rated the Web-based PDR system between fairly easy and very easy to use. Similarly, when using the Web-based viewing of observations feature, all 9 supervisors found it very easy or fairly easy to use. Of those who used the Web-based enrollment and administrative features, 6 (67%) found it very easy or easy to use, with only 1 individual indicating a neutral rating. Of the professionals, 7 (78%) rated the system as much easier than previous tracking methods they had used, with an additional 1 (11%) indicating it was easier and 1 (11%) indicating they were neutral about this method. All professionals indicated they would recommend this system to other programs or foster parents.

Foster parents also reported high levels of satisfaction. Using a 7-point scale from very easy to very difficult, 18 (93%) described the systems as easy or very easy to use. Regarding the system features, 19 (95%) of parents used the Web-based PDR feature and 2 (10%) reported using the automated telephone PDR system (the greater than 100% sum reflects parents who used both methods across the 7 days). All users reported the Web-based PDR was fairly easy, easy, or very easy to use. Only 1 user rated the automated telephone PDR system as fairly difficult. All parents rated the PDR system as somewhat easier, easier or much easier than the previously used tracking methods, and all parents indicated they would either recommend or strongly recommend this system to other foster parents.

### Quality of Video Observation

Recording and viewing the videos of the clinical and foster parent meetings largely involved balancing factors such as frame size, image resolution, frame speed, and audio quality with the intent of making a viewable image that does not make file transfer prohibitive. As the meetings were scheduled for 2 hours and there were generally quite a few people involved, we attempted to push the limits of file size given the current technology and the information technology resources available at average community mental health centers. The research team worked with numerous microsocial coding schemes [21,22] and determined that the video recordings of clinical team and foster parent support group meetings that were made through the system were suitable for fidelity coding for ratings and fidelity checks. While the frame size and image quality would not be suitable for microsocial coding of facial expression and affect, it was reported to be more than suitable for MTFC fidelity coding. The inclusion of a broad-range array microphone during testing appeared to enhance the ability of the system to capture audio content from the numerous individuals in the confined meeting rooms.

With respect to the videos of clinical staff meetings that were recorded for observation, 4 MTFC consultants rated 3 video clips relative to audio and video quality on 5-point scales anchored by 1 = impossible, 3 = codeable, and 5 = easy to code. The video content was rated an average of 4.75 and the audio content an average 4.42. Consultants also indicated a dichotomous (yes/no) response as to whether each segment was codeable, with 100% reporting yes.

## Discussion

We developed a user-friendly and secure Web-based system using state-of-the-art computer-based protocols for recording questionnaire and observational data generated by community-based MTFC staff and foster parents. We are greatly encouraged by the positive satisfaction and utilization results, and we believe these results provide a good evidence base for further evaluation in a randomized controlled trial in future research. Consistent with other studies, our study found that reducing cost and increasing convenience for the user were given as reasons for delivery over the Internet [23]. We believe that these positive results are due to following development guidelines described by other researchers. We followed the methodology used for the development of ClinicalTrials.com as delineated by McCray and colleagues [24]: (1) internal testing by the development team, (2) review by collaborators and consultants, (3) user testing with participants, and (4) measurement of participant Web accessibility. As well, we adapted a development methodology outlined by Ritterband and colleagues [25]: (1) identify the problem area, (2) find an empirically supported intervention, (3) operationalize the treatment, (4) consider the legal issues, (5) design engaging components, (6) tailor them as appropriate, (7) incorporate feedback, (8) develop a Web-based program, and (9) evaluate the program. For future development work, we have high expectations, with Web technology being advanced by the advent of video recording and HTML5 and using these guidelines for development.

For many Internet-based mental health projects, most ethical considerations are limited to issues of security and confidentiality. In this study we followed all secure sockets layer security and institutional review board standards, yet the overall ethical considerations of whether to use technology in this application is still in question. Internet delivery overcomes isolation due to time, mobility, and geographical constraints, but it may not be a substitute for face-to-face contact [23]. For the foster parents’ report of child behavior, there are many anecdotal reports of assistance provided through person-to-person telephone communication using a live PDR caller. In future studies with a larger sample, we will investigate whether the lack of a live caller reduces the opportunity for person-to-person communication.

While the system was created for the specific requirements of MTFC, the usefulness of the system with other validated programs is clear (eg, the ability to provide quick access to securely transmitted clinical session information can help with the remote supervision of therapists from a variety of treatment models). In addition, while the scope of this pilot project called for a demonstration of the feasibility of the system, several foster parents did not want to return to being called for behavioral reports on their foster children, and they continued to enter the information on this Web-mediated system. Increasing the options for foster parents to complete these program requirements appears to have resulted in greater compliance and more consistent completion of the PDR for some foster care providers, a critical aspect for fidelity. These increases in behavioral data will allow program supervisors to more closely match interventions to the current presentation of the involved youth, and to match program reactions to the needs and stress experienced in the foster home, thereby increasing foster parent retention while reducing the response cost for complying with the program requirements. Given that fidelity of these empirically validated treatments has been shown to be related to the quality of their outcomes, it is critically important to develop innovative service delivery systems such as that developed herein that increase fidelity in the dissemination of these proven treatment approaches.

## Abbreviations

HTML: Hypertext Markup Language
ITFMS: Internet-based treatment fidelity monitoring system
MTFC: Multidimensional Treatment Foster Care
PDR: Parent Daily Report

## References

[1] Chamberlain P (2003). Treating Chronic Juvenile Offenders: Advances Made Through the Oregon Multidimensional Treatment Foster Care Model (Law and Public Policy: Psychology and the Social Sciences).

[2] Mihalic S, Irwin K, Fagan A, Ballard D, Elliott D (2004). Successful program implementation: lessons from blueprints. Juv Justice Bull.

[3] Henggeler SW (2004). Decreasing effect sizes for effectiveness studies- implications for the transport of evidence-based treatments: Comment on Curtis, Ronan, and Borduin (2004). J Fam Psychol.

[4] Aos S, Phipps P, Barnoski R, Lieb R (1999). The Comparative Costs and Benefits of Programs to Reduce Crime: A Review of National Research Findings With Implications for Washington State, version 3.0.

[5] Chamberlain P, Reid JB (1991). Using a specialized foster care community treatment model for children and adolescents leaving the state mental hospital. Journal of Community Psychology.

[6] Chamberlain P, Reid JB (1998). Comparison of two community alternatives to incarceration for chronic juvenile offenders. Journal of Consulting and Clinical Psychology.

[7] Eddy JM, Chamberlain P (2000). Family management and deviant peer association as mediators of the impact of treatment condition on youth antisocial behavior. Journal of Consulting and Clinical Psychology.

[8] Elliot DS (1998). Blueprints for Violence Prevention.

[9] US Department of Health and Human Services (1999). Mental Health: A Report of the Surgeon General.

[10] US Department of Health and Human Services (2000). Youth Violence: A Report of the Surgeon General.

[11] Chamberlain P (1998). Treatment foster care. Juv Justice Bull.

[12] Eddy JM, Whaley RB, Chamberlain P (2004). The prevention of violent behavior by chronic and serious male juvenile offenders: A 2-year follow-up of a randomized clinical trial. J Emot Behav Disord.

[13] Chamberlain P, Reid JB (1987). Parent observation and report of child symptoms. Behav Assess.

[14] Baggett KM, Davis B, Feil EG, Sheeber LB, Landry SH, Carta JJ, Leve C (2010). Technologies for expanding the reach of evidence-based interventions: Preliminary results for promoting social-emotional development in early childhood. Topics Early Child Spec Educ.

[15] Feil EG, Baggett KM, Davis B, Sheeber L, Landry S, Carta JJ, Buzhardt J (2008). Expanding the reach of preventive interventions: development of an Internet-based training for parents of infants. Child Maltreat.

[16] Feil EG, Severson H, Taylor T, Bowles S, Webster-Stratton C (2005). Computer based Incredible Years Parent Training for parents of Head Start children: challenges and outcomes.

[17] Taylor TK, Webster-Stratton C, Feil EG, Broadbent B, Widdop CS, Severson HH (2008). Computer-based intervention with coaching: an example using the Incredible Years program. Cogn Behav Ther.

[18] Barrera M, Glasgow RE, McKay HG, Boles SM, Feil EG (2002). Do Internet-based support interventions change perceptions of social support?: An experimental trial of approaches for supporting diabetes self-management. Am J Community Psychol.

[19] Feil EG, Glasgow RE, Boles S, McKay HG (2000). Who participates in Internet-based self-management programs? A study among novice computer users in a primary care setting. Diabetes Educ.

[20] Feil EG, Noell J, Lichtenstein E, Boles SM, McKay HG (2003). Evaluation of an Internet-based smoking cessation program: lessons learned from a pilot study. Nicotine Tob Res.

[21] Davis B, Sheeber LB, Hops H, Reid JB, Patterson GR, Snyder J (2002). Coercive family processesadolescent depression. Reid JB, Patterson GR, Snyder J, editors. Antisocial Behavior in Children and Adolescents: A Developmental Analysis and the Oregon Model for Intervention.

[22] Reid JB, Patterson GR, Baldwin DV, Dishion TJ, Rutter M, Tuma AH, Lann IS (1988). Observations in the assessment of childhood disorders. Rutter M, Tuma AH, Lann IS, editors. Assessment and Diagnosis in Child Psychopathology.

[23] Griffiths F, Lindenmeyer A, Powell J, Lowe P, Thorogood M (2006). Why are health care interventions delivered over the internet? A systematic review of the published literature. J Med Internet Res.

[24] McCray AT, Dorfman E, Ripple A, Ide NC, Jha M, Katz DG, Loane RF, Tse T (2000). Usability issues in developing a Web-based consumer health site. Proc AMIA Symp.

[25] Ritterband LM, Gonder-Frederick LA, Cox DJ, Clifton AD, West RW, Borowitz SM (2003). Internet interventions: in review, in use, and into the future. Prof Psychol Res Pr.

